# A criterion for the reliable use of MRI-only radiotherapy

**DOI:** 10.1186/1748-717X-9-16

**Published:** 2014-01-09

**Authors:** Marie E Korsholm, Line W Waring, Jens M Edmund

**Affiliations:** 1 Department of Oncology, Radiotherapy Research Unit (52AA), Herlev Hospital, University of Copenhagen, Herlev, Denmark; 2 Department of Biomedical Sciences and The Danish National Research Foundation Centre for Cardiac Arrhythmia, Faculty of Health Sciences, University of Copenhagen, Copenhagen, Denmark

## Abstract

**Background:**

MRI-only radiotherapy will eliminate the systematic registration errors introduced when transferring MRI information to the CT. However, challenges concerning the missing information on electron density, necessary for dose calculation and patient setup on bony anatomy are introduced. This study presents a possible statistical approach to evaluate, if deviations based on MRI-only radiotherapy as compared to the CT based radiotherapy are acceptable.

**Methods:**

18 head-and-neck, 21 prostate, 10 vesica and 8 pelvic patients were included in the study. Data from each patient contained a CT and a T2-weighted MRI scan, a structure set and a clinically approved CT based treatment plan, which was re-calculated with identical parameters on the density corrected MRI scans. A statistical analysis including a 95% confidence interval was performed in clinically relevant DVH points.

**Results:**

The mean differences in the investigated DVH points were in the order of 1.5% for the PTV and up to 4.2% for organs at risk. In addition, a proposed criterion of 2% dose difference in the PTV coverage for 95% of the patients was fulfilled for all diagnostic groups for a bulk segmented MRI in the DVH points, D_median_ and D_2%_, while only head-and-neck and prostate further fulfilled the criterion in D_98%_.

**Conclusion:**

Here, we suggested a method for establishing a reliable use of MRI-only radiotherapy. A population-based study comparing CT based dose calculations with those obtained on a suggested segmentation of MRI should be initiated and acceptable deviations in clinically relevant DVH points should be established. Such a population-based approach could form a part of the clinical commissioning of MRI-only radiotherapy.

## Introduction

The gold standard for radiotherapy (RT) planning is computed tomography (CT). However, little discrimination between the soft tissues is obtained from the CT, since tumour and the organs at risk (OARs) have similar attenuation coefficients. Magnetic resonance imaging (MRI) is therefore increasingly combined with CT for a better delineation of the tumour and OARs. MRI has proven beneficial for multiple treatment sites such as head-and-neck (HN), prostate, pelvic and brain [1-4].

Transferring the MRI delineated structures to the CT scan requires a registration between the two modalities. This introduces a systematic registration error arising from deformable anatomical changes and inconsistent patient setup at the MRI and CT scan. For example, different rectal and bladder filling could cause a relative deformation of the prostate, and an imperfect setup of HN patients could result in different bending of the neck. The net result is that (ideally non-distorted) MRI delineations with one relative relationship between structures are transferred to the anatomy of the CT scan with a different relative relationship between the structures on which the treatment is planned. Systematic registration errors have been quantified for prostate approx. 2 mm (average displacement) [5], rectum approx. 2 mm (average displacement) [6] and head 1.8 ± 2.2 mm (average displacement and one standard deviation) [7].

An alternative to the CT based RT is so-called MRI-only RT, where MRI is the only modality in all steps of the treatment workflow. It has been demonstrated that an MRI-only simulation can reduce spatial systematic uncertainties by 2 mm compared to a CT based workflow for prostate patients [8]. MRI-only RT can potentially lead to a simplified workflow reducing workload and cost [9], while easing patient discomfort related to multiple scans especially in palliative cases. Additionally, MRI-only based RT enables the use of co-registered functional MRI for assessment of treatment response and adaptive RT [10], and recently, it introduces the possibility of real-time MRI-guided RT [11]. The main challenges for MRI-only RT are geometrical distortion and the lack of the electron density information needed for dose calculation and setup verification on bone [12].

The dosimetric impact of performing dose calculations on MRI as compared to CT has previously been reported, although these differences are somewhat inconsistently described. Kristensen et al. observed a 2% difference for brain tumours in the prescribed dose region while larger differences were observed for volumes enclosed by lower iso-dose levels [13]. Jonsson et al. reported mean differences in monitor units (MUs) of 0.2% with a standard deviation of 0.5 for the prescription point for different MRI bulk density corrected geometries of prostate and thorax patients [12]. Lambert et al. reached a mean difference of approximately 2% for the prostate using a similar approach [14]. These studies used conventional (non-IMRT) techniques and did not present any quantitative measure to determine whether the differences were acceptable in a more general setting.

With MRI-only based RT, no comparison of the obtained dose distribution with CT is available, and hence, the order of the dosimetric uncertainty for a patient is unknown and might be unacceptable, e.g. for a serial OAR. One way to evaluate the reliability of MRI-only based RT would be to state a 95%-probability that a patient has an acceptable maximum deviation from the CT calculated dose distribution for clinical relevant DVH points or uniform equivalent dose conversions.

Here, we present a statistical framework as a possible tool to decide whether deviations based on MRI-only RT as compared to standard CT are of an acceptable order. To illustrate the method, data from dose calculations on MRI scans as compared to CT are investigated for three DVH points of the PTV as recommended by ICRU Report 83 [15]. The results are reported for multiple treatment sites and techniques.

## Methods

Retrospective data from 18 HN patients (with oro- and hypo-pharyngeal cancer) treated with a static intensity-modulated radiotherapy (IMRT) setup, 21 prostate, 10 vesica and 8 pelvic (not prostate or vesica) patients treated with volumetric modulated arc therapy (VMAT) were included. Each patient had a CT-scan (Philips Big Bore CT), a T2 weighted MRI (Philips 1 T Panorama), an MRI delineated structure set including targets and OARs and a clinically approved treatment plan (Eclipse version 10.0, Varian Medical Systems).

**Figure 1:**
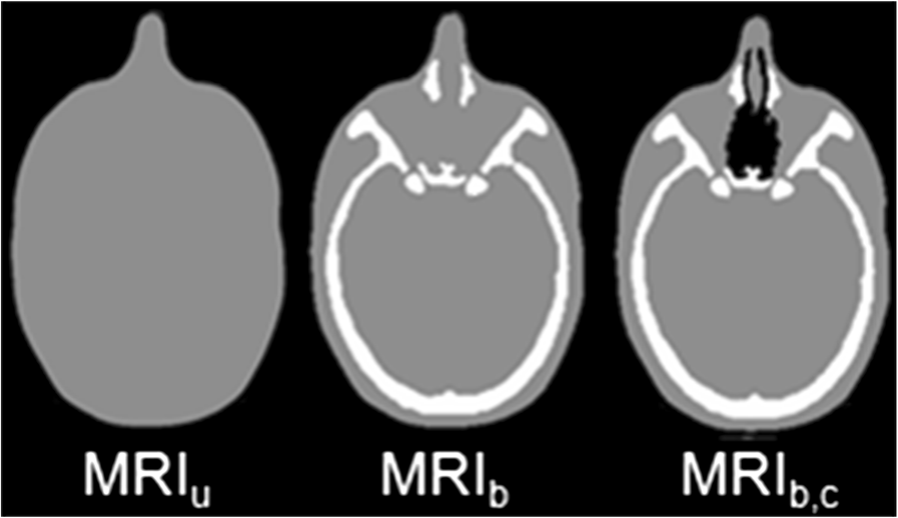
**The different density corrected MRIs for a HN patient.** Left: A homogeneous density assigned MRI. Middle: A bulk density assigned MRI. Right: A bulk density assigned MRI including air cavities.

MRI-only based dose calculations were performed with different uniform density corrections, displayed in Figure 1.

• A homogeneous density assigned MRI (MRI_u_) where the entire body was assigned the electron density of water (Hounsfield unit (HU) =0).

• A bulk heterogeneous density assigned MRI (MRI_b_) where, in addition, the CT delineated bone was transferred to the MRI and assigned a bone specific electron density.

• For the vesica-, prostate- and pelvic patients the assigned electron density was based on the average age of each diagnostic group. For the HN patients, an age-independent electron density was assigned according to skeleton cranium. Table 1 displays an overview of the electron densities and the corresponding calculated HUs.

**Table 1:** The calculated Hounsfield units for bone

**Diagnostic group**	**Bone tissue**	**Electron density [g/cm**^**3**^**]**	**Calculated HU (age)**
HN	Skeleton-cranium (whole)	1.61 (adult)	971
Prostate	Skeleton-femur (whole)	1.33 (30 years)	349 (66.8 years*)
		1.22 (90 years)	
Pelvic	Skeleton-femur (whole)	1.33 (30 years)	356 (64.7 years*)
		1.22 (90 years)	
Vesica	Skeleton-femur (whole)	1.33 (30 years)	309 (78.8 years*)
		1.22 (90 years)	

The bone HUs are calculated based on generic electron densities from ICRU Report 46 [16] and a parameterization of our CT calibration curve from ICRU Report 42 [17]. *The average age in the diagnostic group.

• An additional bulk heterogeneous density assigned MRI (MRI_b,c_) for the HN patients where the effect of air cavities was investigated. Here, CT-delineated air cavities were transferred to the MRI and assigned the electron density equal to that of air. The effect of gas pockets in the bowels was not investigated, since these pockets have an irreproducible interfractional variation in amount and location.

Prior to the MRI-only based dose calculation, the CT based treatment plan and structures (bone and cavity) were registered to the corresponding MRI. The body was outlined separately on the MRI to include possible effects of geometrical distortion. The dose distribution was re-calculated on the density corrected MRI keeping the CT planning parameters, i.e. photon fluence, beam energies, angles, MLC control points, monitor units (MUs) etc. unchanged.

To test if such a CT based re-calculation accurately represents the dose differences present when only MR images are available, 10 prostate treatment plans were optimized on MRI according to our clinical standards and re-calculated on CT with unchanged MRI planning parameters. Subsequently, no significant change in the observed dose difference between the CT- and MRI-planning approaches could be identified for the investigated DVH points. Therefore, the re-calculation on MRI with unchanged CT planning parameters was found to be appropriate.

### DVH point investigation

The CT and MRI based dose distributions were compared in a number of relevant DVH points including the PTV coverage recommended in ICRU Report 83 [15]. The PTV DVH points were the near-maximum absorbed dose (D_2%_), the near minimum absorbed dose (D_98%_) and the median absorbed dose (D_median_). Elective target volumes such as lymph nodes were not included in the investigation. In addition, DVH points for different OARs were compared. The investigated OARs and corresponding DVH points were based on the clinical guidelines used in our department.

### Statistical analysis

We adopt a statistical approach on the dose coverage of a patient cohort as a measure of the reliability of MRI-only RT. We state that 95% of the patients receiving MRI-only RT should have an uncertainty on the dose calculation of the PTV coverage within 2% with respect to that of CT. Such statistical considerations have previously been accepted and incorporated into clinical practice on PTV margin calculations [18]. Ahnesjö et al. presented a table for the individual components contributing to the overall uncertainty of delivering RT [19]. If the uncertainty is 1% and 2% on the CT and MRI dose calculation, respectively, the uncertainty contribution in dose calculation will be 2.2% adding the errors in quadrature. The resulting overall uncertainty would then be 3.3%, which we here considered acceptable.

A statistical analysis comparing the density corrected MRI versus CT based dose calculations for each diagnostic group was carried out to establish 95% confidence intervals (CI) and test the influence of significance (significance level α = 0.05). An univariate analysis of variance (ANOVA) was performed with an one-way two-tailed ANOVA using the statistical software R version 2.11.0 [20]. If the ANOVA displayed a significant difference, a pair wise means comparison was performed with a TukeyHSD analysis for further investigation [21]. The TukeyHSD decreases the risk of detecting a false positive result, i.e. significance, as compared to a t-test. Prior to the statistical analysis, the data were tested and found to be independent and approximately normally distributed with constant variances thereby fulfilling the assumptions of the chosen statistical methods. The dosimetric differences will be presented as percentage difference with respect to CT ± two standard deviations thereby describing the approximate 95% CI of the differences. The CIs were based on the standard deviations calculated from the individual diagnostic groups and not from the pooled data of the ANOVA.

The investigation of rectum for the prostate patients was split into two groups since the prescribed dose showed to be significant in a two-way two-tailed ANOVA.

This is a non-interventional retrospective study assuring the quality of treatment using different image modalities. According to the National Health Research Ethics Committee in Denmark (DNVK), such a study does not require an ethics approval.

## Results

The percentage differences between the DVH points from the CT and the density corrected MRIs are displayed in Table 2. In general, the mean percentage differences for PTV coverage were in the order of 1.5%, which is in agreement with results previously reported. The percentage differences for OARs were up to 4.2% and no statistical difference could be detected for the investigated OARs. In 97% of the investigated DVH points, non-significant differences had p-values above 0.1.

**Table 2:** Statistical results of the DVH point analysis

**Diagnostic group**	**Volume (# of patients)**	**DVH point**	**MRI**_**u **_**[%]**	**MRI**_**b **_**[%]**	**MRI**_**b,c **_**[%]**	**Significance (p-value)**
Prostate	PTV (21)	D_median_	1.3 ± 1.4	−0.0002 ± 1.1	-	A(0.0), B(0.0)
		D_98%_	1.4 ±1.9	−0.03 ± 1.7	-	A(0.01), B(0.0008)
		D_2%_	1.4 ± 1.3	−0.02 ± 1.0	-	A(1.0 · 10^-7^), B(0.0)
	Rectum* (12)	D_10%_	2.0 ± 1.7	0.6 ± 1.6	-	NS
		D_30%_	1.9 ± 2.5	0.8 ± 2.5	-	NS
		D_60%_	1.0 ±3.0	0.2 ± 3.0	-	NS
	Rectum* *(9)	D_10%_	2.2 ± 3.0	0.9 ±3.0	-	NS
		D_30%_	0.8 ± 1.5	−0.008 ± 1.3	-	NS
		D_60%_	−0.06 ± 1.6	−0.7 ± 1.6	-	NS
HN	PTV (18)	D_median_	1.0 ± 1.9	−0.6 ± 1.2	−0.02 ± 3.0	B(0.02)
		D_98%_	1.6 ± 2.3	−0.006 ± 1.8	−1.0 ± 2.8	C(0.005)
		D_2%_	1.2 ± 2.0	−0.4 ± 0.9	0.4 ± 4.3	B(0.04)
	Medulla (18)	D_max_	0.8 ± 3.6	−1.4 ± 3.8	−1.4 ± 3.6	NS
	Parotid sin (16)	D_mean_	−1.2 ± 6.8	2.5 ± 7.4	−1.3 ± 8.7	NS
	Parotid dxt (16)	D_mean_	1.7 ± 7.4	0.2 ± 6.8	1.4 ± 8.9	NS
Vesica	PTV (10)	D_median_	0.4 ± 1.2	−0.3 ± 1.3	-	B(0.008)
		D_98%_	−0.9 ± 4.8	−1.4 ± 4.2	-	NS
		D_2%_	1.1 ± 1.5	0.2 ± 1.8	-	A(0.05)
	Rectum (10)	D_2cm_ ^3^	0.7 ± 1.6	−0.1 ± 1.7	-	NS
		V_40Gy_	−0.1 ± 3.0	−0.6 ± 3.2	-	NS
	Femur sin (10)	D_max_	1.4 ± 3.4	−0.6 ± 1.9	-	NS
	Femur dxt (10)	D_max_	0.7 ± 2.0	−0.7 ± 2.0	-	NS
	Intestine (8)	D_2cm_ ^3^	−0.5 ± 3.5	−0.9 ± 3.4	-	NS
		V_35Gy_	4.2 ± 8.0	3.5 ± 7.6	-	NS
Pelvic	PTV (8)	D_median_	−0.2 ± 1.3	−0.3 ± 1.2	-	NS
		D_98%_	−0.9 ± 2.0	−1.5 ± 2.0	-	NS
		D_2%_	0.8 ± 1.4	0.03 ± 1.6	-	NS
	Femur sin (8)	D_mean_	0.3 ± 1.1	−0.5 ± 1.3	-	NS
		D_max_	0.07 ± 1.5	−0.2 ± 3.3	-	NS
	Femur dxt (8)	D_mean_	0.1 ± 3.1	−0.2 ± 2.2	-	NS
		D_max_	0.3 ± 2.5	−0.4 ± 1.4	-	NS

The percentage differences of MRI_u,_ MRI_b_ and MRI_b,c_ with respect to CT, in mean value ± two standard deviations corresponding to the approximate 95% confidence interval. *Prescribed dose of 70 Gy. **Prescribed dose of 78 Gy.

CT vs. MRI_u_ (A), MRI_u_ vs. MRI_b_ (B), MRI_u_ vs. MRI_b,c_ (C). NS = Non-significant.

CT vs. MRI_b_, CT vs. MRI_b,c_, and MRI_b_ vs. MRI_b,c_ were also tested.

### Prostate patients

**Figure 2:**
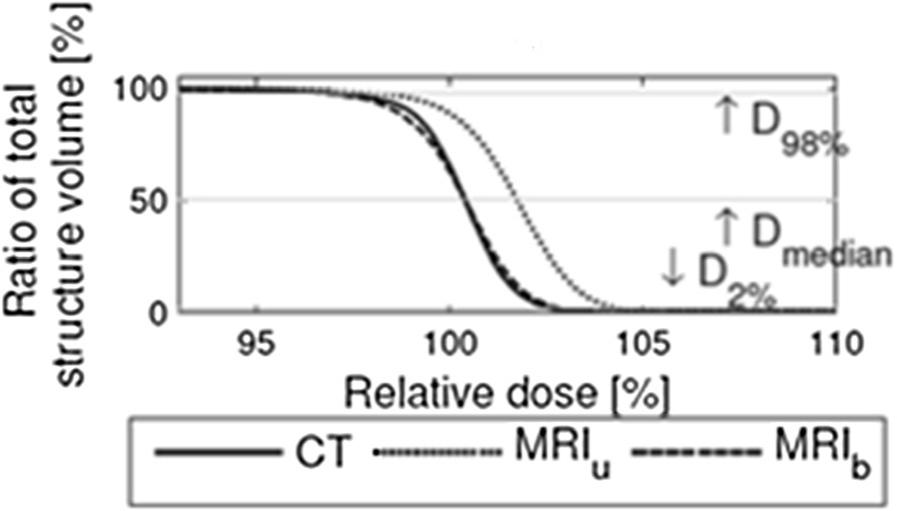
**The average DVH for PTV based on 21 prostate patients.** The intersection between the horizontal lines and the DVHs, is the value of the indicated DVH points.

Investigation of the PTV for the prostate patients showed that MRI_b_ gave results significantly closer to CT than MRI_u_. Figure 2 illustrates the average DVH of the PTV from the 21 prostate patients as well as the investigated PTV DVH points. The differences in the selected DVH points seem to be representative for the differences in the entire DVH.

### Vesica patients

The results for the vesica patients showed that MRI_u_ differed significantly from CT and MRI_b_ in D_median_ and D_2%_ for PTV, while no statistical differences were seen for the remaining investigated DVH points within this diagnosis.

### HN patients

The density corrected MRIs for the PTV in HN patients showed a significant difference between MRI_u_ and CT, and, MRI_b_ and MRI_b,c_ respectively. The bulk density corrected MRIs with and without air cavities did not differ remarkable. No significant differences were seen for the OARs but a large standard deviation for the parotid glands could be observed (see Discussion).

### Pelvic patients

Neither the PTV nor the OARs showed significance when comparing the density corrected MRIs with the CT for the pelvic patients. Large standard deviations, however, were seen for the intestines (see Discussion).

**Figure 3:**
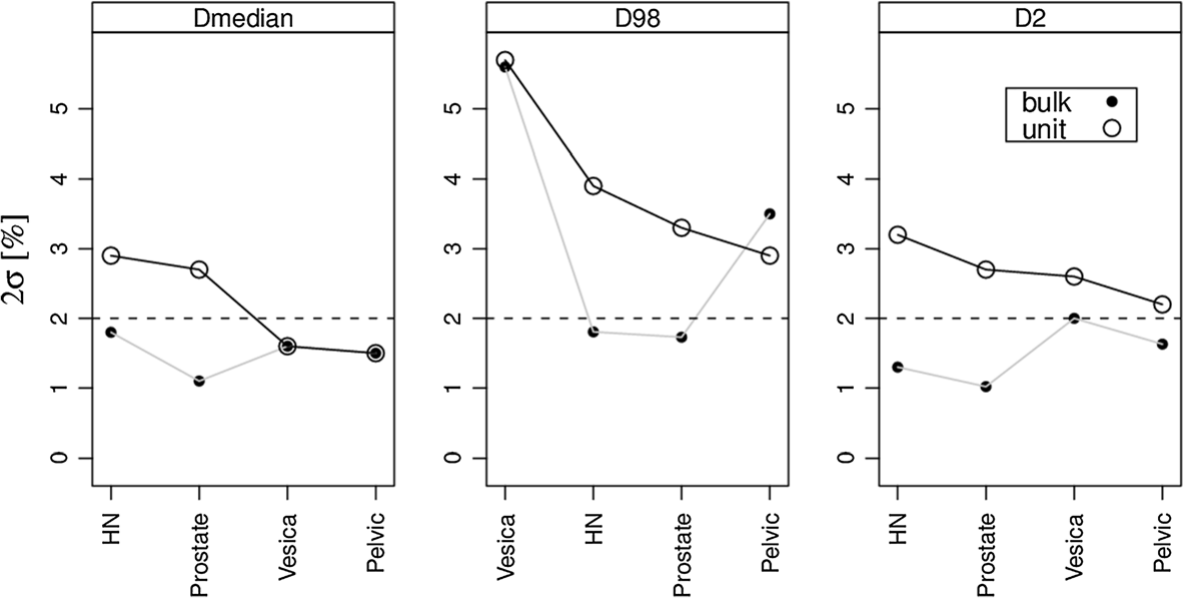
**The approximate 95% CI for the dosimetric differences of the PTV when comparing CT and density corrected MRI.** Deviations are plotted in a descending order for the MRI_u_ data. MRI_u_ = unit. MRI_b_ = bulk.

In Figure 3, conservative 95% confidence intervals are plotted versus diagnosis for the PTV DVH points (PTV coverage). With the bulk density correction for bone, our segmentation criterion of 2% dose calculation deviation was reached for all investigated diagnoses for D_median_ and D_2%_ while only HN and prostate further fulfilled the criterion for D_98%_. With the unit density correction, only the vesica and pelvic patients fulfilled the criteria for the D_median_ point. Hence, one could state that our bulk density correction was sufficient for HN and prostate in MRI-only RT if 95% of the patients should have a dose calculation uncertainty of 2% or less. On the PTV coverage, no clear correlation between a significant difference from CT and passing of the acceptance criteria were found.

## Discussion

MRI-only RT receives increasingly attention as MRI becomes more integrated into modern RT [22,23]. Further, evidence of including MRI into new treatment sites is emerging. Sparing of the hippocampus in cranial irradiation could be one such example that would require an MRI for palliative treatment otherwise traditionally carried out on CT alone [24]. This raises the question of when it is clinically acceptable to perform RT based on MRI as the only modality.

Here, we suggested a method to evaluate the reliability for introducing MRI-only RT. A population based study comparing CT based dose calculations with those carried out on the segmented MRI of choice (e.g. [25]) should be initiated. Acceptable deviations in clinically relevant DVH points should be established. In this study we accepted a 2% deviation in PTV coverage for 95% of the patients and assumed an uncertainty of 1% on the CT based calculations. If a more conservative uncertainty estimate from CT based calculations is assumed, e.g. about 3% as estimated by ICRP more than 10 years ago [26], a more restricted uncertainty requirement should be imposed on the MRI deviation ultimately to reach an overall uncertainty of about 4% [19]. We obtained a conservative CI for the MRI_b_ medulla of 4.2%, which could be critical near tolerance doses for myelopathy. Tighter acceptance criteria, e.g. a 1% deviation for a 99% CI could be considered on normal tissue complication probabilities that receive a high priority.

The size of the patient cohort will influence the statistical power and therefore the credibility by which a segmentation strategy fulfill a criterion for MRI-only RT. The size of the patient cohort included in the study can influence the ability to estimate a dosimetric difference with acceptable statistical power. A power analysis on the available CT dose statistics in the relevant DVH points should therefore be carried out. For example, the D_98%_ mean and standard deviation for the prostate patients CT based dose calculation were 1.94 and 0.03 Gy, respectively. With 21 patients, the ability to detect a mean difference of at least 2% could be made with a power of 96%. As seen in the middle panel of Figure 3, such a calculation did not indicate whether a segmentation criteria for MRI-only RT was fulfilled for 95% of the patients (bulk passes and unit does not) but only support the credibility of the statistical analysis. A limitation in the proposed approach was that only a measure of similarity in the DVH points was described without any spatial information of where the differences were located.

Large CIs of 8 and 7% were found for the intestines and parotid glands, respectively. The former could be due to irreproducible gas pockets, which were not transferred to the MRI from CT, and were considered less critical. For the latter, inspections of the CT and MRI showed that the main differences in the body-outline were in the area around the ears, which potentially influenced the calculated dose to the parotid glands. Therefore, this area needs special attention during MRI acquisition and should possibly be corrected for geometrical distortion. Generally, OARs are in lower dose regions and small differences in absolute dose would appear as larger percentage differences. Therefore, the standard deviations in general were found to be larger for the OARs than the PTV DVH points.

The investigation of the HN patients showed similar results when comparing the bulk density corrected MRIs with and without air cavities, this may be related to the fact that no patients with nasopharyngeal cancer, where the dose distributions are expected to be more influenced by air cavities, were included. Hence, the effect of air cavities in nasopharyngeal cancer patients should be investigated further.

For our bulk density correction the majority of the patients showed an uncertainty of 2% or less, on the PTV coverage. The patients that did not fulfil the criteria and their corresponding dose distribution were investigated. For the D_98%_, 1 prostate (1/21), 1 HN (1/18), 2 vesica (2/10) and 2 pelvic (2/8) patients had deviations above 2% which is consistent with the diagnosis passing the criteria in the middle panel of Figure 3. For the abdominal area, these patients had multiple gas pockets leading to dose valleys in the corresponding MRI dose distributions. The position of the tumor bed of the (post-surgery) HN patient was close to the trachea. This was uncorrected for in the MRI_b_ segmentation with a percentage deviation of 2.3% whereas the deviation was 0.6% in the MRI_b,c_ segmentation. These findings could speak in favor of including air segmentation for all diagnostic groups although this was not the strategy chosen in this study. One vesica patient did not meet the criteria D_2%_, which could be explained by an abnormally large change in body outline on MRI as compared to CT due to an anatomic deformation (interfractional variation).

This study separated the results for the different diagnostic groups why the impact of the different delivery techniques could not be estimated, as each diagnostic group was treated with the same delivery technique. Since our main results are consistent with those reported previously obtained with 3D conformal RT, the impact is expected to be minimal.

## Conclusion

We have suggested a method for establishing a reliable use of MRI-only radiotherapy. A population-based study comparing CT based dose calculations with those obtained on a suggested segmentation of MRI should be initiated and acceptable deviations in clinically relevant DVH points should be established. Such a population-based approach could form a part of the clinical commissioning of MRI-only radiotherapy.

## Competing interest

On behalf of all authors, the corresponding author states that there is no conflict of interest.

## Authors’ contribution

JME conceived the study. JME and MEK drafted the manuscript. MEK and LWW collected and analyzed the data. All authors read and approved the final manuscript.
